# Quarterly Report of Proceedings

**Published:** 1858-10

**Authors:** 


### QUARTERLY REPORT OF THE PROCEEDINGS OF THE EPIDEMIOLOGICAL SOCIETY.

---

On Monday, July 5th.

JOHN PROPERT, Esq., Vice-President, in the Chair.

### PAPER READ.

"On the Difficulties Attending the Study of Epidemic Diseases." By W. H. Michael, Esq., M.R.C.S., of Swansea.

Discussed by Dr. Milroy, Dr. Murchison, Dr. Greenhow, and Mr. Radcliffe.

### DONATIONS TO THE LIBRARY OF THE SOCIETY.

1. "Health and Meteorology of the Metropolis," for June, July, and August." Presented by the General Board of Health.
2. "Report on the Sanitary Condition of the Whitechapel District, for the three months ending July 3rd, 1858." By John Liddle, Esq. Presented by the Author.
3. "Purification of the Thames." A letter by F. O. Ward, Esq. Addressed to William Coningham, Esq., M.P. Presented by the Author.

### MEETING.

The opening Meeting of the Session 1858-9 will be held on Monday, November 1st, at No. 37, Soho Square, when, after a short introductory address by Dr. Babington, a Paper will be read by Dr. Barker, of Bedford.

